# Molecular Insights into the Genesis of Heat Hardening in Marine Bivalves

**DOI:** 10.3390/antiox14121468

**Published:** 2025-12-07

**Authors:** Ioannis Georgoulis, Ioannis A. Giantsis, Basile Michaelidis, Athanasios Kouniakis, Konstantinos Feidantsis

**Affiliations:** 1 Laboratory of Animal Physiology, Department of Zoology, School of Biology, Aristotle University of Thessaloniki, GR-54124 Thessaloniki, Greece; michaeli@bio.auth.gr; 2 Laboratory of Ichthyology & Fisheries, Faculty of Agriculture, Forestry and Natural Environment, Aristotle University of Thessaloniki, GR-54124 Thessaloniki, Greece; igiants@auth.gr; 3 Department of Fisheries & Aquaculture, School of Agricultural Sciences, University of Patras, GR-26504 Mesolonghi, Greece; athanasioskouniakis@gmail.com

**Keywords:** Mediterranean mussel, hardening genes, antioxidant defense, heat shock response, mitochondrial metabolism, oxidative stress, cell death responses, autophagy, gene expression, mRNA levels

## Abstract

Heat hardening induces complex biochemical reprogramming that enhances thermal resilience in marine bivalves. Despite this technique’s promising results in marine animals, the molecular basis of heat hardening is far from understood. This study elucidates the molecular mechanisms underlying the hardening process in *Mytilus galloprovincialis* exposed to a 4-day sublethal heat treatment. Induction of *hsf-1*, *hsp70*, and *hsp90* genes revealed the activation of the heat shock response and proteostasis machinery, ensuring proper protein folding and preventing oxidative and proteotoxic stress. Simultaneous upregulation of mitochondrial (*atpase6*, *cox1*, *nadh*) and glycolytic (*pk*, *cs*) genes reflects enhanced oxidative phosphorylation and glycolytic flux, maintaining ATP supply and metabolic flexibility under elevated temperatures. Increased *hif-1α* expression suggests transient hypoxia signaling, coordinating oxygen utilization with redox control. Reinforcement of antioxidant defenses, together with elevated autophagy-related transcription, denotes a shift toward oxidative stress mitigation and damaged organelle clearance. Balanced expression of pro- (*bax*) and anti-apoptotic (*bcl-2*) factors, along with *nf-κb* modulation, supports tight regulation of cell survival and inflammatory responses. These findings underscore a highly integrated biochemical network linking proteostasis, intermediary metabolism, redox balance, and antioxidant defense with cellular quality control, which together underpin the physiological plasticity of heat-hardened *M. galloprovincialis*, enhancing survival under transient thermal stress.

## 1. Introduction

Marine organisms are increasingly exposed to thermal stress due to ongoing climate change, which poses significant risks for their survival, distribution, and ecological function. The Mediterranean mussel *Mytilus galloprovincialis* (Lamarck, 1819) is particularly vulnerable to rising seawater temperatures, as heat stress can disrupt cellular homeostasis, decrease energy availability, and ultimately increase mortality. However, thermal tolerance in mussels is a plastic trait that can be modulated through acclimation to different thermal regimes, as well as through brief exposures to sublethal temperatures, known as heat hardening [1,2,3].

Heat hardening is recognized as a form of stress priming or hormesis, whereby mild stress exposures induce a transient physiological “memory” response that enhances resistance to subsequent challenges and more severe stressors [4]. Thus, in regard to global warming, it confers improved heat tolerance to elevated temperature [1,2]. Mitochondria have been identified as central control organelles of hormesis, which activate a series of protective responses when organisms are exposed to mild stresses [5]. Such responses include the production of reactive oxygen species (ROS) and antioxidant enzymes, and heat shock protein (Hsp) induction [6], which in turn activates cytoprotective autophagic responses [7,8]. More specifically, antioxidant defense and Hsps are both crucial cellular defense mechanisms that work together to protect against cellular damage caused by oxidative stress. Antioxidant enzymes are the first line of defense that contributes to the dynamic equilibrium between ROS production and detoxification [9], while Hsps maintain the proper structure and function of antioxidant enzymes and other vital proteins, also contributing to the protection of cells from ROS [10]. This coordinated interaction helps maintain protein homeostasis and ameliorate apoptosis and inflammation, thereby strengthening the overall resilience and enhancing cell survival of the organism [11]. Our previous work has demonstrated that laboratory-induced heat hardening substantially improves the resilience of *M. galloprovincialis* under thermal stress. Heat-hardened mussels exhibited enhanced electron transport system (ETS) activity, greater antioxidant capacity, and decreased mortality rates at elevated temperatures, compared to non-hardened populations [12]. Subsequent metabolomic and gene expression analyses revealed diversification of metabolic pathways, increased tricarboxylic acid (TCA) cycle activity, and the accumulation of metabolites with cytoprotective roles, such as taurine and formate. These findings suggested that heat hardening facilitates energy reallocation and supports redox balance during thermal stress [13,14]. Further studies highlighted the importance of cellular pathways such as autophagy, apoptosis, and inflammation, showing that hardened mussels maintain higher energy availability and enhanced autophagic performance, while dampening apoptosis and inflammatory responses under heat stress [15]. Importantly, field validation demonstrated that these physiological benefits extend to natural conditions, where hardened mussels retained stress memory and sustained thermal tolerance across seasonal changes and increasing seawater temperatures [16]. This inference ranks heat hardening of mussels as an extremely valuable tool that can be applied against climate change effects, which can be catastrophic for mussel culture.

Hence, heat hardening is considered to trigger a broad suite of protective physiological responses, ranging from energy metabolism and redox regulation to protein homeostasis, antioxidant defense, and cellular survival pathways. The cellular mechanisms underlying this process are thought to involve the coordinated activation of multiple stress signaling pathways, including reactive oxygen, nitrogen, and carbonyl species signaling, the unfolded protein response, and transcription factor activation, ultimately leading to increased resistance [17,18,19,20], but, in fact, the underlying biochemical mechanisms, particularly those underlying the “stress memory” effect that extends protection beyond the immediate exposure, remain, to a great extent, unexplored, let alone in bivalves. In the present study, we aimed to examine the molecular mechanisms underlying the development of Mediterranean mussel hardened phenotypes following a 4-day laboratory heat hardening process. For this reason, we examined how prior exposure of mussels to heat hardening creates a precondition state that can induce coordinated molecular adaptations, including antioxidant defense, heat shock protein expression, mitochondrial metabolism, apoptosis, and inflammation. According to previous results, our hypothesis was that the heat hardening method enhances expression of a variety of genes related to these pathways, which leads to faster, stronger, and more sensitive reactions when mussels are later exposed to more severe heat stress. By focusing on cellular pathways that regulate stress tolerance, we seek to elucidate how short-term preconditioning enhances the ability of mussels to withstand thermal challenges, thereby providing new insights into the adaptive capacity of marine bivalves in the face of climate change.

## 2. Materials and Methods

### 2.1. Animals

*M. galloprovincialis* mussels, with a mean (±SD) weight of 27.82 ± 3.85 g, length of 6.56 ± 0.34 cm, and width of 3.42 ± 0.13 cm, were collected from a commercial mussel farm in Vistonikos Gulf, North Aegean Sea, Greece, when the ambient seawater temperature was approximately 18 °C. The mussels were transported to the Laboratory of Animal Physiology, Department of Zoology, School of Biology, Aristotle University of Thessaloniki, and maintained in 1000 L tanks with recirculating, aerated natural seawater for one week.

During acclimation, mussels were fed daily with cultured microalga *Tisochrysis lutea* (CCAP 927/14) at 0.5% of their total body weight. Water temperature was maintained at 18 °C ± 0.5, salinity at 34‰ ± 2.85, and pH at 8.12 ± 0.05.

### 2.2. Experimental Design

#### 2.2.1. Laboratory Exposure—Hardening Process

**Figure 1:**
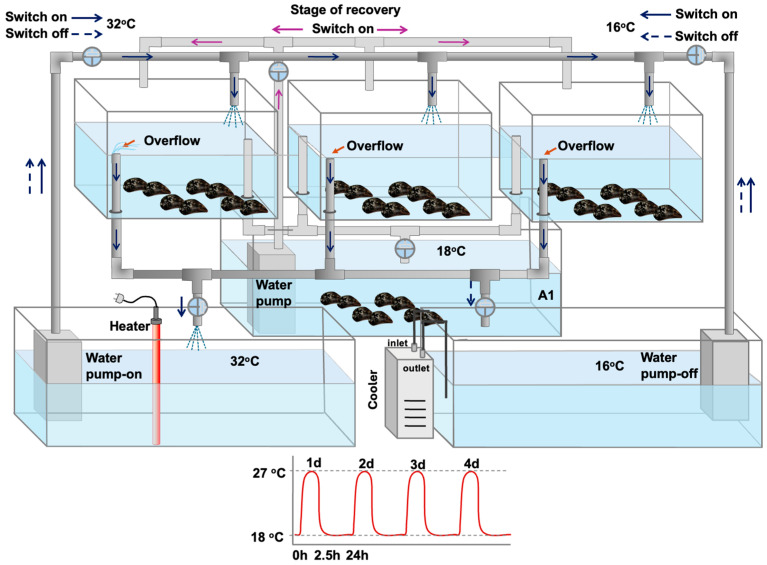
Mussel heat hardening process in which mussels underwent four consecutive 2.5 h heat-stress bouts (27 °C), followed by a 24 h recovery period (18 °C): tanks with fully aerated water at 18 °C were connected with switches with two other tanks, one with increased (32 °C) and the other with decreased (16 °C) water temperature, in order to regulate the water temperature to increased (27 °C) or decreased (18 °C—control) temperatures, as fully described and depicted in Georgoulis et al. [12].

Under laboratory conditions, mussels were exposed to two distinct phases: a hardening phase and a non-hardening phase. The hardening phase followed Hutchison’s “Repeated Critical Thermal Maximum (CTM)” method [21], with treatments applied as previously described by Georgoulis et al. [12] (2021). In short, mussels underwent four consecutive heat-stress bouts. Each bout consisted of a 2.5 h thermal shock, during which the temperature increased from 18 °C to a sublethal 27 °C at a rate of 1.5 °C per min, followed by a 24 h recovery period at 18 °C (1.5 °C per min) (Figure 1).

In contrast, mussels in the non-hardening phase were not subjected to these treatments and were continuously kept at 18 °C in identical tanks. Mussels exposed to the hardening protocol are hereafter referred to as hardened (H), while those kept at a constant 18 °C are referred to as non-hardened (NH) (Figure 1).

#### 2.2.2. Tissue Collection

After each of the four consecutive heat-stress bouts, both H and NH animals (*n* = 5) were collected. Immediately after, animals were dissected and mantle tissues were collected, immediately embedded in Eppendorf tubes, frozen in liquid nitrogen, and transferred to the laboratory, where they were stored at −80 °C until further analysis. Mantle tissue was chosen because it exhibits higher aerobic capacity and more intense physiological stress responses compared to other tissues like posterior adductor muscle [22]. Furthermore, its selection aligns with our previous studies on heat hardening in *Mytilus galloprovincialis*, thereby providing a consistent basis for comparative molecular and biochemical analysis.

### 2.3. Determination of Gene Expression at the mRNA Level

RNA was isolated from 50 mg of mantle tissue using the NucleoZOL reagent (Macherey-Nagel, Düren, Germany) following the manufacturer’s protocol. The total RNA concentration was measured with a Q5000 spectrophotometer (Quawell Technology, Inc., San Jose, CA, USA), and 50–100 ng of RNA was subsequently used for cDNA synthesis with the PrimeScript 1st Strand cDNA Synthesis Kit (Takara Bio, Kusatsu, Japan).

Quantitative real-time PCR (qPCR) was carried out on an Eco 48 qPCR cycler (PCRmax, Cole-Parmer North America, Vernon Hills, IL, USA) using 10 μL reaction volumes and the KAPA SYBR FAST qPCR Master Mix (2X) (KAPA Biosystems, Wilmington, MA, USA). Primers for Caspase2, Caspase3, Caspase8, Bcl-2, Bax, NF-κB, Hsp70, COX1, ND-2, AOX, and Atp6 were designed as previously reported [12,15] (Table 1). Amplification products were verified by electrophoresis on 1.5% agarose gels. Relative gene expression levels were calculated using the actin gene as a reference, following the methodology described in our earlier work [12].

### 2.4. Statistics

Changes in the examined genes’ relative mRNA levels were analyzed for significance at the 5% level (*p* < 0.05) using one-way or two-way analysis of variance (ANOVA), depending on the number of factors considered, with GraphPad Instat 3.0 and GraphPad Prism 5.0, respectively. Sampling days and treatment (hardening vs. non-hardening) were treated as fixed factors. Post hoc comparisons were conducted using the Bonferroni test. Significant main effects of seasonality and treatment, as well as their interactions, were observed (*p* < 0.05).

Principal component analysis (PCA) was performed with FactoMineR package in R (The R Project for Statistical Computing, version 4.3.2) [30] to identify patterns of correlated variables. Data are presented as means ± standard deviation (SD) for *n* = 5.

## 3. Results

### 3.1. Heat Shock Response

**Figure 2:**
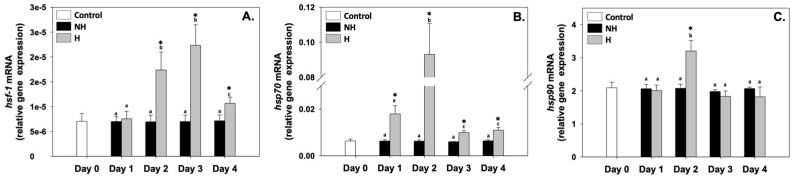
Changes in the relative *hsf-1* (**A**), *hsp70* (**B**), and *hsp90* (**C**) mRNA levels in the mantle of *Mytilus galloprovincialis* mussels during the 4-day heat hardening process. The values are presented as the means ± SDs of *n* = 5. Lower-case letters denote *p* < 0.05 differences between mussels of the same group (H or NH), while an asterisk (*) denotes *p* < 0.05 differences compared to the control (day 0) and between H and NH mussels on the same day.

Heat shock factor 1 (*hsf-1*), heat shock protein 70 (*hsp7*0), and heat shock protein 90 (*hsp9*0) relative mRNA levels in the NH mussels remained unchanged between exposure days, as well as compared to the control on day 0 (Figure 2), whereas *hsf-1* relative mRNA levels in the H mussels significantly increased (compared to the respective NH mussels) mainly on day 2 and day 3, and thereafter decreased on day 4 (Figure 2A). The *hsp70* and *hsp90* of the H mussels exhibited their highest relative mRNA levels mainly on day 2, thereafter decreasing (Figure 2B,C). While *hsp70* relative mRNA levels in the H mussels significantly increased compared to the NH mussels on all 4 days of the process (Figure 2B), *hsp90* relative mRNA levels between H and NH mussels significantly differed only on day 2, and remained unchanged on day 1, day 3, and day 4 of the hardening process (Figure 2C).

### 3.2. Intermediate Metabolism

**Figure 3:**
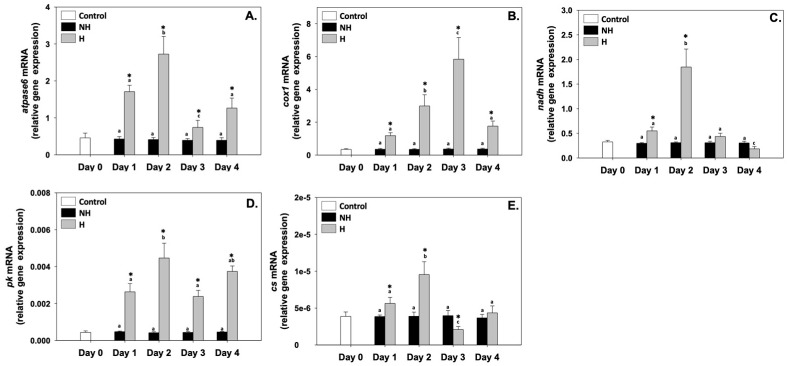
Changes in the relative *atpase6* (**A**), *cox1* (**B**), *nadh* (**C**), *pk* (**D**), and *cs* (**E**) mRNA levels in the mantle of *Mytilus galloprovincialis* mussels during the 4-day heat hardening process. The values are presented as the means ± SDs of *n* = 5. Lower-case letters denote *p* < 0.05 differences between mussels of the same group (H or NH), while an asterisk (*) denotes *p* < 0.05 differences compared to the control (day 0) and between H and NH mussels on the same day.

ATPase6 (*atpase6*), cytochrome c oxidase I (*cox1*), NADH dehydrogenase (*nadh*), pyruvate kinase (*pk*), and citrate synthase (*cs*) relative mRNA levels in the NH mussels remained unchanged between exposure days, as well as compared to the control on day 0 (Figure 3). Regarding *atpase6*, its relative mRNA levels in the H mussels significantly increased compared to the NH mussels on all 4 days of the process, with the highest levels observed on day 1 and even more so on day 2 (Figure 3A). Regarding *cox1*, its relative mRNA levels in the H mussels also remained significantly increased compared to the NH mussels on all 4 days of the process, and a progressively increasing pattern was observed peaking on day 3, which thereafter decreased (Figure 3B). *nadh* and *cs* exhibited a similar pattern: their relative mRNA levels in the H mussels significantly increased (compared to the respective NH mussels), mainly on day 1 and day 2, and thereafter decreased on day 3 and day 4 (Figure 3C and Figure 3E, respectively). *pk* relative mRNA levels in H mussels remained significantly increased compared to the NH mussels on all 4 days of the process, with the highest levels observed mainly on day 2 and day 4 of the process (Figure 3D).

### 3.3. Hypoxia

**Figure 4:**
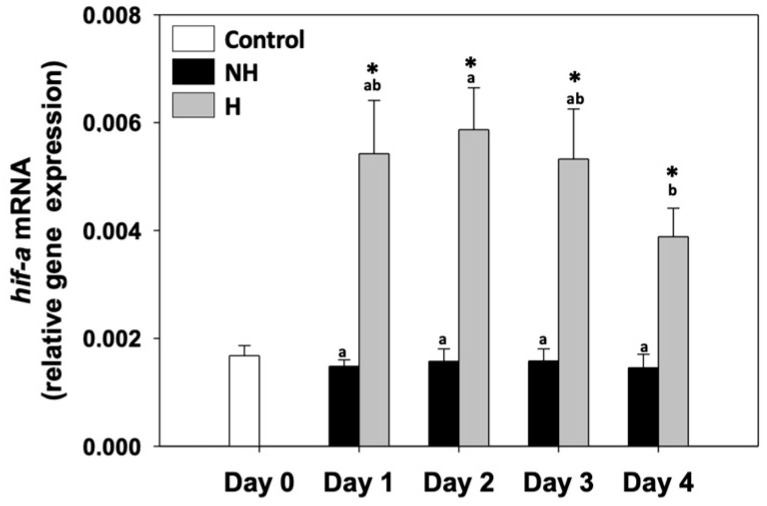
Changes in the relative *hif1-a* mRNA levels in the mantle of *Mytilus galloprovincialis* mussels during the 4-day heat hardening process. The values are presented as the means ± SDs of *n* = 5. Lower-case letters denote *p* < 0.05 differences between mussels of the same group (H or NH), while an asterisk (*) denotes *p* < 0.05 differences compared to the control (day 0) and between H and NH mussels on the same day.

Hypoxia induced factor 1α (*hif1-a*) relative mRNA levels in the NH mussels remained unchanged between exposure days, as well as compared to the control on day 0. *hif1-a* relative mRNA levels in H mussels remained significantly increased compared to the NH mussels on all 4 days of the process, with the highest levels observed mainly on day 2 and the lowest on day 4 of the process (Figure 4).

### 3.4. Antioxidant Defense

**Figure 5:**
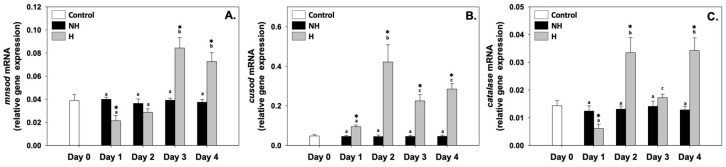
Changes in the relative *mnsod* (**A**), *cusod* (**B**), and *catalase* (**C**) mRNA levels in the mantle of *Mytilus galloprovincialis* mussels during the 4-day heat hardening process. The values are presented as the means ± SDs of *n* = 5. Lower-case letters denote *p* < 0.05 differences between mussels of the same group (H or NH), while an asterisk (*) denotes *p* < 0.05 differences compared to the control (day 0) and between H and NH mussels on the same day.

Mn superoxide dismutase (*mnsod*), Cu superoxide dismutase (*cusod*), and catalase (*catalase*) relative mRNA levels in the NH mussels remained unchanged between exposure days, as well as compared to the control on day 0 (Figure 5). *mnsod* relative mRNA levels in the H mussels significantly increased (compared to the respective NH mussels) mainly on day 3 and day 4, while on day 1, these levels significantly decreased compared to the respective NH mussels, and on day 2, they were unchanged (Figure 5A). *cusod* relative mRNA levels in H mussels remained significantly increased compared to the NH mussels on all 4 days of the process, with the highest levels observed mainly on day 2, the lowest levels on day 1, and intermediate levels on day 3 and day 4 of the process (Figure 5B). Regarding *catalase*, its relative mRNA levels in the H mussels significantly increased compared to the NH mussels on day 2 and day 4; on day 1, these levels significantly decreased, while on day 3, they were unchanged compared to the respective NH mussels (Figure 5C).

### 3.5. Autophagy and Apoptosis

**Figure 6:**
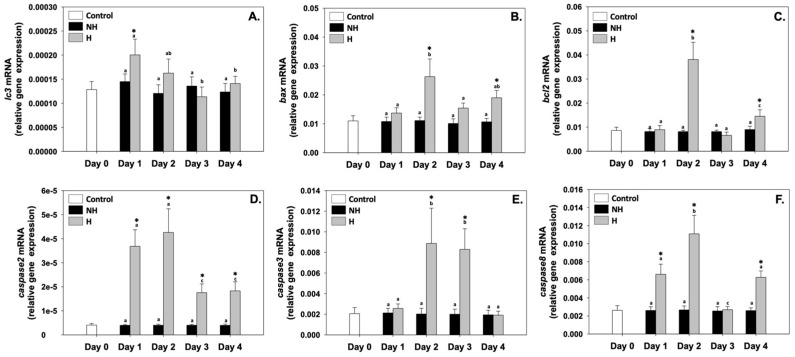
Changes in the relative *lc3b* (**A**), *bax* (**B**), *bcl-2* (**C**), *caspase2* (**D**), *caspase3* (**E**), and *caspase8* (**F**) mRNA levels in the mantle of *Mytilus galloprovincialis* mussels during the 4-day heat hardening process. The values are presented as the means ± SDs of *n* = 5. Lower-case letters denote *p* < 0.05 differences between mussels of the same group (H or NH), while an asterisk (*) denotes *p* < 0.05 differences compared to the control (day 0) and between H and NH mussels on the same day.

Microtubule-associated protein 1 light chain 3 beta (*lc3b*), Bcl-2 associated-X protein (*bax*), B-cell lymphoma 2 (*bcl-2*), caspase 2 (*caspase2*), caspase 3 (*caspase3*), and caspase 8 (*caspase8*) relative mRNA levels in the NH mussels remained unchanged between exposure days, as well as compared to the control on day 0 (Figure 6). Regarding *lc3b*, its relative mRNA levels in the H mussels significantly increased compared to the NH mussels only on day 1, while during the rest of the days, these levels were unchanged compared to the NH mussels (Figure 6A). *bax* and *bcl-2* relative mRNA levels in the H mussels significantly increased compared to the NH mussels on day 2 (exhibiting the highest levels) and day 4 of the process, while on day 1 and day 2, they were similar to those of NH mussels (Figure 6B and 6C, respectively). *caspase2* relative mRNA levels in H mussels significantly increased compared to the NH mussels on all 4 days of the process, with the highest levels observed mainly on day 2 and day 4 of the process (Figure 6D). *caspase3* relative mRNA levels in H mussels significantly increased compared to the NH mussels only on day 2 and day 3, while on day 1 and day 4, they were unchanged compared to the NH mussels (Figure 6E). Regarding *caspase8* relative mRNA levels in the H mussels, a progressively increasing pattern was observed, peaking on day 2, which thereafter decreased to NH levels on day 3 and again increased on day 4 compared to the respective NH mussels (Figure 6F).

### 3.6. Inflammation

**Figure 7:**
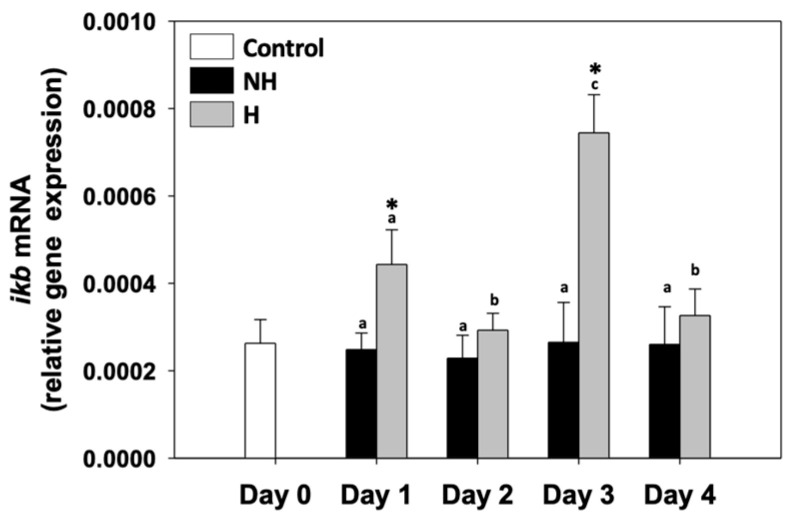
Changes in the relative *ikb* mRNA levels in the mantle of *Mytilus galloprovincialis* mussels during the 4-day heat hardening process. The values are presented as the means ± SDs of *n* = 5. Lower-case letters denote *p* < 0.05 differences between mussels of the same group (H or NH), while an asterisk (*) denotes *p* < 0.05 differences compared to the control (day 0) and between H and NH mussels on the same day.

Inhibitor of kappa B (*ikb*) relative mRNA levels in the NH mussels remained unchanged between exposure days, as well as compared to the control on day 0. *ikb* relative mRNA levels in the H mussels significantly increased compared to the NH mussels on day 1 and day 3, while on day 2 and day 4, they were unchanged compared to the respective NH mussels (Figure 7). 

### 3.7. Multivariate Analysis

**Figure 8:**
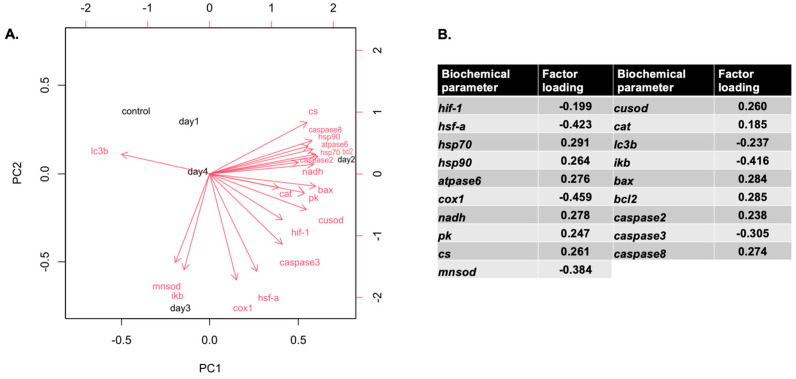
Correlations of variables with the first two principal components (PCs) from the multivariate analysis (**A**), along with a table summarizing the contributions of the biochemical parameters based on factor loadings (**B**). The PCA was derived from the full biochemical and physiological dataset. Parameters indicated by red vector arrows were used as predictors in building the PCA.

*hsp70*, *hsp90*, *atpase6*, *nadh*, *pk*, *cs*, *cusod*, *catalase*, *bax*, *bcl-2*, *caspase2*, and *caspase 8* mRNAs were positively correlated with PC1, and *lc3b* mRNA was negatively correlated with PC1, while *hif-1*, *hsf-a*, *cox1*, *mnsod*, *ikb*, and *caspase3* mRNAs were negatively correlated with PC2. Overall, 59.51% of the variance was attributed to PC1, while 23.04% was attributed to PC2. Cumulatively, PC1 and PC2 explain 82.55% of the total variance in the dataset. Most of the mRNAs (apart from *lc3b* mRNA) formed a clear cluster with day 2 and day 3 of the heat hardening process (Figure 8).

## 4. Discussion

The present study aims to elucidate the induction of a coordinated molecular and biochemical response in *M. galloprovincialis* when exposed to a short-term heat hardening protocol, which subsequently enhances thermal tolerance and cellular resilience. By examining key pathways—including the heat shock response (HSR), intermediary metabolism, hypoxia signaling, antioxidant defense, autophagy, apoptosis, and inflammation—this work provides mechanistic insight into how mussels integrate multiple cellular processes to cope with elevated temperatures. Importantly, the temporal dynamics of gene expression reveal that heat hardening does not merely induce isolated stress responses, but establishes a biochemically interconnected network, supporting the concept of “stress memory” in marine ectotherms.

### 4.1. Heat Shock Response and Proteostasis

Heat hardening elicited HSR in *M. galloprovincialis*, with *hsf-1*, *hsp70*, and *hsp90* upregulation (mainly on days 2 and 3, Figure 2), indicating an early and transient activation of the proteostasis machinery. Hsps are highly conserved molecular chaperones that play a central role in maintaining protein homeostasis under stress by facilitating protein refolding, preventing aggregation of denatured proteins, and targeting irreversibly damaged proteins for proteasomal degradation [12,31]. Moreover, Hsp70 regulates apoptosis through interactions with Bax and caspases, while Hsp90 stabilizes proteins involved in signaling pathways such as NF-κB, linking protein quality control to stress and immune responses [32,33]. Their activity also extends to the stabilization of metabolic enzymes, allowing continued ATP generation and redox balance even under thermally destabilizing conditions [10,34,35]. The temporal increase in Hsp expression on days 2–3 suggests that proteostatic mechanisms are maximally engaged after initial stress signaling, thereby laying the biochemical foundation for downstream metabolic adjustments, antioxidant responses, and cell survival pathways. Both *hsp70* and *hsp90* relative mRNA expression levels in mussels show an increase from 22 °C, indicating their ability to sense thermal stress, even before the respective cellular processes are initiated [22]. Therefore, they can be considered among the earliest molecular indicators activated during heat hardening [12,16], demonstrating a highly plastic response and suggesting their important role in the regulation of thermal plasticity [36]. Comparable HSR dynamics have been widely reported in bivalves and other ectotherms exposed to sublethal thermal stress, supporting the universality of chaperone-mediated cytoprotection as a first line of defense that enables subsequent physiological adaptations [1,34].

### 4.2. Intermediary Metabolism: Coordinating Energy Supply

*atpase6*, *cox1*, *nadh*, *cs*, and *pk* upregulation in H mussels (Figure 3, Figure 4 and Figure 5) reflects a coordinated enhancement of both mitochondrial oxidative phosphorylation and glycolytic flux, ensuring adequate ATP supply during elevated thermal demand. ATPase6 and Cox1 are essential for maintaining proton motive force and thus ATP synthesis, while NADH dehydrogenase activity secures electron transfer from the TCA cycle to the electron transfer system (ETS) [37]. In parallel, *cs* upregulation highlights increased TCA cycle activity, providing both reducing equivalents (NADH, FADH_2_) and metabolic intermediates necessary for energy metabolism and biosynthesis. The increased levels of *pk* expression (Figure 3) suggest that glycolysis is simultaneously intensified, providing a rapid source of ATP and precursors for anabolic pathways, a strategy often adopted under thermal stress when energy turnover is high [38].

Increased Hsp activity likely preserves the structural integrity and activity of these enzymes, allowing sustained metabolic flux despite proteotoxic stress [13,14]. The temporal increases in mitochondrial gene expression levels on days 2–3 correspond closely to increased Hsp induction, suggesting a biochemical cross-talk between proteostasis and metabolism. This metabolic adjustment not only provides continuous ATP production but also helps to control ROS levels by maintaining balanced electron flow through the ETS, decreasing the risk of electron leakage. Such integration of glycolysis, the TCA cycle, and oxidative phosphorylation seems to provide the energetic and redox foundation required to support downstream biochemical responses, such as antioxidant defense, and regulation of the autophagic and apoptotic cell death responses. Preconditioning mussels through the heat hardening protocol appears to induce stress memory, enabling faster and stronger activation of these genes when the mussels encounter future extreme environmental temperatures [12,13,15,16].

### 4.3. Hypoxia Signaling: Oxygen Sensing Under Elevated Metabolism

Elevated mitochondrial activity in H mussels probably increases cellular oxygen demand, which in turn activates the transcriptional regulator Hif-1α (Figure 4). Hif-1α, which serves as a central oxygen sensor, stabilizes under hypoxic conditions, and in turn induces the expression of genes that favor anaerobic glycolysis while downregulating oxygen-intensive metabolic processes [39]. This shift allows cells to maintain ATP production when oxygen supply may not match the heightened demand of an activated mitochondrial system. In addition, Hif-1α regulates antioxidant and survival pathways by decreasing the oxidative damage associated with mitochondrial ROS overproduction [40].

The overlap between *hif1-a*’s and mitochondrial genes’ relative mRNA levels suggests a feedback mechanism, where oxygen-sensing pathways act to balance energy metabolism with redox stability. By promoting glycolytic ATP supply and modulating mitochondrial respiration, mussels may avoid excessive ROS leakage while ensuring sufficient energy availability. This mechanism is consistent with the metabolic flexibility concept, which indicates that organisms adjust aerobic and anaerobic pathways depending on environmental and physiological demands [34]. Therefore, the transient upregulation of *hif1-a* in H mussels reflects not only a compensatory mechanism to meet increased energy demands, but probably a protective strategy for the maintenance of cellular homeostasis under conditions of elevated thermal stress.

### 4.4. Antioxidant Defense: Redox Homeostasis

The activation of mitochondrial metabolism and Hif-1α pathways during heat hardening increases ROS production, requiring a robust antioxidant defense. In this context, H mussels’ *mnsod*, *cusod*, and *catalase* differential expression highlights the importance of redox balance in sustaining cellular homeostasis (Figure 5). The delayed induction of *mnsod* on days 3 and 4 suggests that mitochondrial ROS accumulation occurs progressively during sustained metabolic activation, requiring subsequent reinforcement of antioxidant capacity [14,41]. However, the upregulation of *cusod* across all days of heat hardening reflects its broader role in superoxide radical detoxification. This early and sustained activation indicates that cytoplasmic ROS buffering is critical from the onset of stress exposure. Meanwhile, *catalase* showed a more variable expression pattern, with peaks on days 2 and 4. This suggests a complementary role to SOD enzymes, fine-tuning ROS detoxification according to cellular redox demands.

The observed antioxidant responses are consistent with the fact that moderate ROS levels serve as signaling molecules to activate protective pathways, while excessive ROS would trigger oxidative distress and cellular damage [42]. The response to ROS probably represents a hormetic response [43]. Early ROS suppression is a strategy by which cells tightly control ROS production to maintain redox balance and avoid excessive oxidative stress, then followed by controlled ROS increases that act as signals to initiate adaptation processes [43]. In this framework, the coordination of timing and magnitude of *mnsod*, *cusod*, and *catalase* expression seems to maintain ROS within a functional window that promotes stress adaptation without compromising macromolecular integrity in H mussels. This signaling safeguards proteins, lipids, and DNA but also intersects with other stress-response pathways, including apoptosis, autophagy, and inflammation, representing a critical node linking metabolic activation with downstream survival mechanisms. In H mussels, the combined enhancement of HSR and antioxidant defenses forms a preparatory strategy that reinforces protection against more intense subsequent thermal episodes [12,44].

### 4.5. Autophagy and Apoptosis: Cellular Quality Control

Heat hardening modulated the autophagic and apoptotic pathways, which serve as cellular quality-control mechanisms, acting in a delicate balance to determine cell fate under stress. *lc3b* upregulation on day 1 in H mussels (Figure 6) suggests an early activation of autophagy, likely as a rapid protective response to eliminate damaged organelles and misfolded proteins produced during the initial metabolic and oxidative challenge [45]. By recycling macromolecules, autophagy seems to provide metabolic substrates that support ATP production, necessary for the increased mitochondrial activity observed during the same period. Autophagy induction likely represents an early and transient adaptive response during initial heat-stress exposure; however, its activity may decline or be modulated as the hardening response stabilizes. As heat exposure persisted, the increase in pro-apoptotic markers such as *bax* and *caspases* (*2*, *3*, *8*) on days 2 and 4 indicated activation of apoptotic signaling in H mussels, suggesting the need for irreparably damaged cells, while the concurrent increase in the anti-apoptotic *bcl-2* highlights a compensatory mechanism aimed at restricting excessive cell loss [46]. This tuning between pro- and anti-apoptotic markers probably plays a beneficial role in improving thermal tolerance that allows H mussels to selectively eliminate vulnerable cells while preserving tissue integrity. As shown from previous research by Georgoulis et al. [15,16], although H mussels exhibited increased pro-apoptotic gene expression levels, they accumulated significantly lower levels of both Bax and cleaved caspases at elevated temperatures compared to NH mussels, indicating delayed activation and decreased levels of cell death pathways. Furthermore, *hsp70* overexpression was observed, also serving as an inhibitor of apoptosis by preventing the activation and activity of caspases [47], while the increased expression levels of *bcl-2* have also been associated with inhibition [48]. By integrating autophagy with apoptosis, H mussels probably sustain cellular homeostasis under thermal stress. This strategy minimizes energy waste, preserves functional cells, and contributes to the overall survival advantage observed in H individuals compared to NH ones [15,16].

The parallel induction of apoptotic genes, Hsp expression, antioxidant activation, and Hif-1α signaling suggests a cross-talk between proteostasis, redox balance, and cell death pathways. For instance, we could hypothesize that Hsps inhibit apoptosome formation and stabilize mitochondrial membranes [49], since apoptotic activity in H mussels was elevated but not uncontrolled. Under the same prism, ROS can activate both autophagy and apoptosis depending on concentration and context [50], underscoring the central role of redox regulation in orchestrating cell fate.

### 4.6. Inflammation: Integrating Cellular Responses

Inflammatory signaling also showed modulation in H mussels, as fluctuations in *ikb* expression (Figure 7) suggest NF-κB activation, a central regulator of immune and inflammatory responses. NF-κB activation under thermal stress can be triggered by ROS and apoptotic signals, linking inflammation directly with the earlier-described antioxidant and apoptotic responses [51]. By promoting transcription of cytokines and survival factors, NF-κB provides an additional layer of defense, ensuring rapid cellular adaptation to damage signals. Interestingly, inflammatory activation was not sustained across all days of the hardening process: on days 2 and 4, *ikb* remained unchanged, suggesting that prolonged NF-κB signaling was actively restrained. This regulation is critical, as excessive inflammation would lead to tissue damage and energy drain, counteracting the benefits of stress adaptation. The coincidence of decreased inflammation with increased autophagy and antioxidant defense probably points to a coordinated regulatory mechanism: autophagy can suppress NF-κB activation by degrading inflammatory mediators, while antioxidants limit ROS-driven inflammatory cascades [52,53]. Moreover, apoptotic regulation seems to be intertwined with inflammation in H mussels, as controlled apoptosis likely prevents the release of intracellular danger signals (damage-associated molecular patterns—DAMPs), thereby limiting unnecessary inflammatory responses [54]. In this context, the moderate upregulation of caspases alongside the restrained inflammatory profile highlights a strategy of damage containment, where cellular turnover occurs without triggering uncontrolled immune activation.

### 4.7. Integrative Perspective: Stress Memory as a Biochemical Network

Heat hardening primes Mediterranean mussels through a temporally dynamic cross-linked biochemical network providing a mechanistic basis for stress memory and enhanced thermal tolerance: Hsp-mediated proteostasis enables efficient metabolism, which supports ATP production and ROS signaling; Hif-1α integrates oxygen sensing with metabolic and antioxidant responses; autophagy and apoptosis maintain cellular quality; and inflammation is transiently modulated to prevent collateral damage. These findings advance understanding of bivalve stress physiology, highlighting the molecular and cellular plasticity that supports survival under fluctuating thermal environments [1,4,12,13,14,15,16,33,39,55].

The observed increase in gene expression levels on days 2–3 likely reflects a coordinated, temporally optimized stress response. On day 1, initial signaling events seem to prime the cells, but transcriptional and translational machinery has not yet reached maximal activity. By days 2–3, HSR, metabolic activation, antioxidant defenses, and autophagic pathways are fully engaged, reflecting the integration of multiple protective mechanisms. The repeated heat shocks and recovery phases during the heat hardening process likely provide the necessary stimulus for mussels to elevate mRNA levels and progressively develop a hardened precondition state. The observed clustering of gene expression changes allows transcriptional and biochemical adaptations to consolidate thermal tolerance. The subsequent decline on day 4 may result from negative feedback, energy conservation, action of synthetized proteins, or partial mitigation of stress, as cells achieve a new homeostatic balance. Such temporal dynamics are consistent with the hormetic nature of heat hardening, where a transient stress induces a strong but time-limited protective response [12,13,14,15,16,55].

### 4.8. Study Limitations

(1) All analyses were conducted exclusively on mantle tissue. Although this is consistent with our previous work on heat hardening [12,13,14,15] and is highly relevant to stress responses, it may not fully capture tissue-specific variation across the whole organism. (2) Our study primarily focused on mRNA levels without quantifying protein levels and post-translational modifications or conducting functional assays (e.g., measuring enzyme activities, LC3B lipidation, and Hif-1α stabilization). Therefore, future studies should also address these post-translational factors.

## 5. Conclusions

Heat hardening primes *M. galloprovincialis* through a temporally orchestrated and biochemically integrated response, whereby Hsp-mediated proteostasis, enhanced metabolic flux, hypoxia signaling, antioxidant defense, autophagy, and controlled apoptosis collectively maintain cellular integrity and promote organismal survival under thermal stress. Antioxidant defense particularly seems to be directly related to mussels’ survival and is triggered by thermal response. Specifically, it seems to modulate ROS to limit cellular damage while promoting protective signaling, and together with autophagy, it prevents damage and maintains cellular homeostasis. Furthermore, HSR possibly acts as an early and essential trigger that stabilizes protein homeostasis and supports antioxidant enzyme activity. These pathways interact temporally, with early Hsp induction and ROS modulation setting the stage for later metabolic adjustments and cellular quality-control mechanisms. The transient modulation of inflammation further underscores the coordination of these pathways. These findings advance our understanding of the molecular and biochemical plasticity of bivalves and provide a mechanistic framework for stress memory, highlighting how brief, sublethal thermal exposures can enhance resilience to subsequent heat challenges. Protective mechanisms such as HSR, antioxidant defense, and autophagy play a crucial role in establishing stress memory during mussel hardening, enhancing resilience both throughout preconditioning and upon subsequent exposure to extreme temperatures. Future work should explore the interaction of these pathways at the protein and metabolite levels to fully elucidate the physiological basis of thermal adaptation in marine organisms.

## Figures and Tables

**Table 1:** Primer nucleotide sequences for the genes amplified in the present study.

Gene	Sequence (5′-3′)	Accession Number	Reference
*atpase6* F	5′-GGTTGTCCGTTAATCCTTGTG-3′	NC006886	Georgoulis et al. [15]
*atpase6* R	5′-AACCAACCCACTACCAACTC-3′		
*caspase2* F	5′-ACAAGTGCAGATGCTGTGTTG-3′	HQ424449.1	Falfushynska et al. [23]
*caspase2* F	5′-ACACCTCTCACATTGTCGGC-3′		
*caspase3* F	5′-ACGACAGCTAGTTCACCAGG-3′	HQ424453.1	Falfushynska et al. [23]
*caspase3* R	5′-CCACCAGAAGAGGAGTTCCG-3′		
*caspase8* F	5′-AATGTCGGTACCCCACGATG-3′	HQ424450.1	Falfushynska et al. [23]
*caspase8* R	5′-CGTGTATGAACCATGCCCCT-3′		
*bcl-2* F	5′-CGGTGGTTGGCAAGGATTTG-3′	KC545829.1	Falfushynska et al. [23]
*bcl-2* R	5′-CGCCATTGCGCCTATTACAC-3′		
*bax* F	5′-TAACTGGGGACGTGTAGGCA-3′	KC545830.1	Falfushynska et al. [23]
*bax* R	5′-CCAGGGGGCGACATAATCTG-3′		
*ikB* F	5′-TGTCATTTGCCGATTCTACGA-3′	MglkB2_Fs	The present study
*ikB* R	5′-GGCTCCATTCCTCCTTAGTG-3′	qlkB2MgR	
*cox1* F	5′-GTGTCTTCTTATGGGTCTG-3′	FJ890849	Woo et al. [24]
*cox1* R	5′-GCTATAAACATGCTTTCTCC-3′		
*nadh* F	5′-TGGTGTTTTCCTCTACACTC-3′	FJ549901	Woo et al. [24]
*nadh* R	5′-AGGGTCTTATTACCCGCACT-3′		
*hsp70* F	5′-CGGAGGCAAGCCAAAACTAC-3′	AB180909.1	Giannetto et al. [25]
*hsp70* R	5′-AGCCTCGGCAGTTTCTTTCA-3′		
*hsp90* F	5′-GGTTGCTGATAAAGTAGTTG-3′	AJ586906.3	Woo et al. [26]
*hsp90* R	5′-ATTCAGTCTGGTCTTCTTTC-3′		
*hsf-1* F	5′-TGGGTAACGGAGCAGCAGA-3′	XM034457664.2	The present study
*hsf-1* R	5′-CATGGGTGGTAGGTTGGATAAG-3′		
*hif-a* F	5′-TGCTAAATACCTTGGCATCTCA-3′	KP185351.1	Giannetto et al. [27]
*hif-a* R	5′-GCTCTCCAAACGGCAATGTA-3′		
*pk* F	5′-GACATGRTTTTYGCSTCCTTCA-3′	XM022456018	Georgoulis et al. [13]
*pk* R	5′-TCATCATCTTCTGKGCVAGGAA-3′		
*cs* F	5′-AACCACGTGACGACCATGCTGAA-3′	LOC143064983	The present study
*cs* R	5′-TCCTCGTAGACATGCTCCCA-3′		
*mnsod* F	5′-GATGCAGCAGTAGCAGTCCA-3′	JN863295.1	Wang et al. [28]
*mnsod* R	5′-GTAGGCATGCTCCCAGACAT-3′		
*cusod* F	5′-AGGCGCAATCCATTTGTTAC-3′	JN863295.1	Wang et al. [28]
*cusod* R	5′-CATGCCTTGTGTGAGCATCT-3′		
*catalase* F	5′-CTCTGACCGTGGAACCCCTGA-3′	AY743716.2	Giannetto et al. [25]
*catalase* R	5′-ATCACGGATGGCATAATCTGGA-3′		
*lc3b* F	5′-CCWCAAGARCTCTCYATGTC-3′	XM_011417532.3	Papadopoulos et al. [29]
*lc3b* R	5′-TCYTGTGAKGCATAWGTCAT-3′	XM_063570666.1	

## Data Availability

Data is contained within the article.

## Abbreviations

The following abbreviations are used in this manuscript:

ETS	Electron transfer system
TCA	Tricarboxylic acid
ROS	Reactive oxygen species
Hsps	Heat shock proteins
CTM	Critical thermal maximum
H	Hardened
NH	Non-hardened
ANOVA	Analysis of variance
SD	Standard deviation
*hsf-1*	Heat shock factor 1
*hsp70*	Heat shock protein 70
*hsp90*	Heat shock protein 90
*atpase6*	ATPase 6
*cox1*	Cytochrome c oxidase I
*nadh*	NADH dehydrogenase
*pk*	Pyruvate kinase
*cs*	Citrate synthase
*hif1-a*	Hypoxia-induced factor 1α
*mnsod*	Mn superoxide dismutase
*cusod*	Cu superoxide dismutase
*lc3b*	Microtubule-associated protein 1 light chain 3 beta
*bax*	Bcl-2-associated X protein
*bcl*	B-cell lymphoma 2
*ikb*	Inhibitor of kappa B
PCA	Principal component analysis
HSR	Heat shock response
DAMPs	Damage-associated molecular patterns
